# RecA Protein Plays a Role in the Chemotactic Response and Chemoreceptor Clustering of *Salmonella enterica*

**DOI:** 10.1371/journal.pone.0105578

**Published:** 2014-08-22

**Authors:** Albert Mayola, Oihane Irazoki, Ignacio A. Martínez, Dmitri Petrov, Filippo Menolascina, Roman Stocker, José A. Reyes-Darias, Tino Krell, Jordi Barbé, Susana Campoy

**Affiliations:** 1 Departament de Genètica i de Microbiologia, Universitat Autònoma de Barcelona, Bellaterra (Cerdanyola del Vallès), Spain; 2 ICFO-Institut de Ciències Fotòniques, Castelldefels, Spain; 3 Department of Civil and Environmental Engineering, Massachusetts Institute of Technology, Cambridge, Massachusetts, United States of America; 4 Department of Environmental Protection, Estación Experimental del Zaidín-Consejo Superior de Investigaciones Científicas, Granada, Spain; Institut National de la Recherche Agronomique, France

## Abstract

The RecA protein is the main bacterial recombinase and the activator of the SOS system. In *Escherichia coli* and *Salmonella enterica* sv. Typhimurium, RecA is also essential for swarming, a flagellar-driven surface translocation mechanism widespread among bacteria. In this work, the direct interaction between RecA and the CheW coupling protein was confirmed, and the motility and chemotactic phenotype of a *S*. Typhimurium *ΔrecA* mutant was characterized through microfluidics, optical trapping, and quantitative capillary assays. The results demonstrate the tight association of RecA with the chemotaxis pathway and also its involvement in polar chemoreceptor cluster formation. RecA is therefore necessary for standard flagellar rotation switching, implying its essential role not only in swarming motility but also in the normal chemotactic response of *S*. Typhimurium.

## Introduction

RecA is a DNA-dependent ATPase [1], [2] present in almost all members of the Domain Bacteria [3], [4]. As a protein that is highly conserved among bacterial species, it is commonly used in phylogenetic studies [5]. In addition, RecA is the main bacterial recombinase involved in the central steps of homologous recombination and recombinational DNA repair [6]–[8]. It is also the activator of the DNA damage response known as the SOS system [9]. In this system, RecA acts as a DNA damage sensor by binding to single-stranded DNA, which activates the protein and thereby its co-protease activity. Activated RecA (RecA*) prompts autocleavage of the LexA repressor, thus inducing its expression and that of other SOS genes, mostly those involved in DNA recombination and repair [9]. RecA* is also able to induce the autocleavage of other serine proteases such as UmuD [10] and several repressors of the bacteriophage lytic cycle [11]–[13]. Furthermore, RecA is directly associated with other repair pathways such as the activated error-prone DNA polymerase V [10], [14], excision base repair [15], and RecN, involved in the repair of double-stranded DNA breaks [16].

Increasing its activity profile, a new role for RecA in swarming motility, has been added to its functional catalog [17], [18]. Swarming is a specialized and highly coordinated form of flagellar-driven multicellular surface translocation [19], [20] and the fastest mode of bacterial surface navigation [21]. In the absence of RecA, the swarming ability of both *Escherichia coli* and *Salmonella enterica* sv. Typhimurium is impaired [17], [18]; interestingly, the same phenotype is observed in *S.* Typhimurium strains overexpressing RecA protein [18]. Thus, none of the as yet known RecA activities, i.e., SOS response activation, recombinational DNA repair, or genetic recombination, seem to be necessary for the control of swarming motility [17]. Nevertheless, a possible link between RecA and the chemotaxis pathway through the CheW protein has been suggested. The CheW coupling protein is essential for the formation of the ternary signaling complex that also contains the CheA autokinase and MCPs (methyl-accepting chemotaxis proteins) [22]. The *in vitro* interaction of RecA and CheW was shown in a large-scale genome-wide screen assay [23] and a balance between the intracellular concentrations of these two proteins was shown to be essential for swarming [18].

Swarming is not governed by chemotaxis, nevertheless it has been described that the chemosensory pathway is essential for the motility on solid surfaces [24]. Mutants in the chemotaxis (*che*) pathway fail to swarm because of defective colony hydration [25], which in turn is associated with the flagellar rotation bias present in *che* mutants. Thus, in the wild-type strain flagellar rotation switches from clockwise (CW) to counterclockwise (CCW), whereas *che* mutants have CW or CCW biases depending on the specific mutation [26]. During CCW rotation, all flagella of the bacterium form a bundle that falls apart when one or more flagellar motors turn in the CW direction. This switch promotes lubrication of the cell–surface interface, which is needed to overcome surface friction, both of which are critical requirements for swarming motility in temperate swarmers such as *S.* Typhimurium and *E. coli* [27]. The impaired swarming phenotype of *che* mutants can be restored by adding water or osmolytes to the semi-solid surface [25], [27] or by restoring the normal flagellar rotation bias [28].

To further investigate the role of RecA in the control of swarming motility, in this work we examined the relationship between RecA and CheW in two-hybrid experiments and on co-immunoprecipitation assays, which confirmed the interaction of these two proteins. In addition, we explored the possibility that RecA affects the bacterial flagellar rotation pattern, by studying the swimming profile, flagellar motor rotation, and the chemotactic response of a *recA* knockout *S.* Typhimurium mutant (*ΔrecA*). The results confirmed a role for RecA in polar chemoreceptor cluster formation and therefore in flagellar rotation switching.

## Materials and Methods

### Bacterial strains, plasmids, and growth conditions

The bacterial strains and plasmids used in this study are listed in Table 1. Except when indicated, all strains of bacteria were grown at 37°C in Luria–Bertani (LB) broth or on LB plates. When necessary, ampicillin (100 µg/ml) or chloramphenicol (34 µg/ml) was added to the culture. The same cell growth conditions were used for the microfluidics and optical trap assays (described below). The bacteria were grown overnight in 2 ml of LB broth supplemented, when needed, with the appropriate antibiotic. Each culture was diluted 1∶10 into LB broth without antibiotics but containing an oxygen scavenging system consisting of 100 µg glucose oxidase/ml and 20 µg catalase/ml (final concentrations) [29]. The added glucose is a substrate for the oxygen scavenging system and provides the energy needed for swimming under anaerobic conditions [29]. The cells were incubated at 37°C for 1 h and then diluted 100-fold in measuring medium (1% Bacto Tryptone, 0.8% NaCl, 2% glucose, 100 mM Tris-Cl, pH 7.5) containing the oxygen scavenging system. In all cases the scavenging system was added at least 2 h before the medium was used, to ensure a stable low level of oxygen.

**Table 1:** Bacterial strains and plasmids used in this work.

Strain or plasmid	Relevant characteristic(s)	Source or reference
**Strains**		
DH5α	*E. coli supE4 ΔlacU169 (φ80 ΔlacZ ΔM15) hsdR17, recA1, endA1, gyrA96, thi-1, relA1*	Clontech
MC1061	*E. coli* F^−^ *Δ*(*ara-leu*)*7697 [araD139] B/r Δ(codB-lacI)3 galK16 galE15 λ^−^ e14^−^ mcrA0 relA1 rpsL150* (Str^R^) *spoT1 mcrB1 hsdR2(r^−^m^+^)*	CGSC
BL21 (DE3) pLysS	*E. coli F^−^ dcm ompT lon hsdS(r_B_^−^m_B_^−^) galλ(DE3)* carring pLysS plasmid, Cm^R^	Stratagene
LT2	*S.* Typhimurium wild type strain	ATCC
UA1907	*S.* Typhimurium *ΔcheWΩcat*. Cm^R^	This work
UA1908	*S.* Typhimurium *ΔcheW*	This work
UA1910	*S.* Typhimurium *ΔcheR*	This work
UA1915	*S.* Typhimurium *ΔcheR ΔcheW*	This work
UA1913	*S.* Typhimurium *ΔcheR ΔrecA*	This work
UA1927	*S.* Typhimurium *ΔrecAΩ,cat.* Cm^R^	This study
UA1928	*S.* Typhimurium *ΔcheB*	[29]
UA1929	*S.* Typhimurium *ΔcheY*	[29]
UA1930	*S.* Typhimurium *ΔcheW ΔrecA*	This work
UA1931	*S.* Typhimurium *ΔrecA*	This work
**Plasmids**		
pKOBEGA	Vector containing the λ Red recombinase system, Amp^r^, temperature sensitive	Generous gift of Prof. G. M. Ghigo, [33]
pKD3	Vector carrying FRT-Cm construction, Amp^R^, Cm^R^	[31]
pCP20	Vector carrying FLP system, OriV_ts_, Amp^R^	[31]
pGEM-T	Cloning Vector; Amp^R^	Promega
pGEX 4T-1	Expression vector carrying the Ptac IPTG - inducible promoter and the *lacI^q^* gene; GST fusion tag, Amp^R^	Amersham Biosciences
pUA1108	pGEX 4T-1 derivative plasmid carrying without the GST fusion tag, carrying only the Ptac promoter and the lacIq gene; used as overexpression vector, Amp^R^	This work
pUA1109	pUA1108 derivative containing the native *S.* Typhimurium *recA* gene under the control of the Ptac promoter, Amp^R^.	This work
pUA1127	pUA1108 derivative vector carrying the eYFP::*cheR* fusion, Amp^R^	This work
pUA1130	pUA1108 derivative containing the native *S.* Typhimurium *recA* gene under the control of the Ptac promoter, Amp^R^.	This work
pUA1131	pUA1108 derivative overexpression vector carrying the *cheW*-FLAG gene	This work
pB2H*Δ*α	pACYCDuet-1 derivative vector with the *E. coli Δ*α β-galactosidase fragment under the control of the Ptac promoter	BCCM/LMBP, [36]
pB2H*Δ*ω	pACYCDuet-1 derivative vector with the *E. coli Δ*ω β-galactosidase fragment under the control of the Ptac promoter	BCCM/LMBP, [36]
pUA1114	pB2H*Δ*α derivative vector contaning the *Δ*α-*recA* fusion	This work
pUA1115	pB2H*Δ*ω derivative plasmid contaning the *Δ*ω-*recA* fusion	This work
pUA1116	pB2H*Δ*α derivative vector contaning the *Δ*α-*cheW* fusion	This work
pUA1117	pB2H*Δ*ω derivative plasmid contaning the *Δ*ω-*cheW* fusion	This work
pUA1118	pB2H*Δ*α derivative plasmid contaning the *Δ*α-*amyA* fusion, used as a control in two hybrid assays.	This work
pUA1119	pB2H*Δ*ω derivative plasmid contaning the *Δ*ω-*dnaE* fusion, used as a control in two hybrid assays.	This work

Dead cells used for optical trap assays were prepared by the addition of 2% formaldehyde to the culture, with subsequent dilution steps carried out following the same protocol used for live bacterial cultures.

For chemotactic assays, overnight cultures of *S.* Typhimurium were grown in tryptone broth (1% Bacto Tryptone, 0.5% NaCl) supplemented, when required, with the appropriate antibiotics [30]. All strains used in the chemotactic assays (LT2, UA1928 (*ΔcheB*), UA1931 (*ΔrecA*) and UA1931/pUA1130) had similar growth kinetics in tryptone broth medium (data not shown). The cultures were then diluted 1∶100 in the same medium but without antibiotics and incubated at 30°C with constant shaking until an OD_600_ of approximately 0.5 was reached. The culture was then harvested by centrifugation at 4500 g for 10 min at room temperature. The obtained cell pellet was washed twice in 1 ml of tempered tethering buffer (10 mM potassium-phosphate pH 7, 67 mM NaCl, 10 mM Na-lactate, 0.1 mM EDTA, and 0.001 mM l-methionine) and the resuspended cells were diluted to approximately 6×10^7^ colony-forming units (cfu)/ml.

### Construction of the *S.* Typhimurium mutant strains

The *S.* Typhimurium mutants were constructed using the one-step PCR-based gene replacement method [31], except when indicated. All DNA techniques were performed as described elsewhere [32]. The chloramphenicol resistance cassette from the plasmid pKD3 was amplified using suitable oligonucleotides containing 80-nucleotide stretches homologous to each of the insertion sites and the corresponding P1 and P2 sites of pKD3 (Table S1). The PCR products were digested with *Dpn*I and used to transform *S.* Typhimurium electrocompetent cells containing the pKOBEGA plasmid [33]. Following selection of the transformant clones, the latter plasmid was eliminated by taking advantage of its temperature sensitivity, incubating the clones at 42°C. Gene substitution was confirmed by PCR and sequencing using the appropriate primers.

To construct the *ΔcheRΔcheW* and *ΔcheRΔrecA* mutant strains, the chloramphenicol resistance cassette present in the *ΔcheR* strain was eliminated as previously described using the pCP20 plasmid [31]. Afterwards, either *ΔrecA* or *ΔcheW* mutations from UA1927 and UA1907 were transferred to the *ΔcheR* chloramphenicol-sensitive strain (UA1910) by transduction, using the P22 HT bacteriophage [34]. The same procedure was used to construct the *ΔrecA ΔcheW* strain. In this case, the chloramphenicol resistance cassette present in *ΔcheW* strain was removed and the *ΔrecA* mutation was transducted from the UA1927 strain. In all cases, the absence of the prophage in the selected clones was determined by streaking them onto green plates as previously described [35]. All of the resulting strains were verified by PCR and sequencing.

### β-Galactosidase-based two-hybrid system

The two-hybrid assay was performed as described [36]. The *recA* and *cheW* genes were PCR-amplified using suitable oligonucleotides (Table S1) that included *Sph*I and *Bam*HI restriction sites in the amplicon. After their release from the plasmids by endonuclease digestion, the amplified genes were cloned in both pB2H*Δ*α and pB2H*Δ*ω vectors. The same procedure was used to clone the *amyA* and *dnaE* genes into pB2H*Δ*α and pB2H*Δ*ω, respectively. These constructs served as the non-interaction assay controls, as previously described [36]. All of the constructs were confirmed by sequencing.

To simultaneously express the two fusion proteins within a cell, electrocompetent *E. coli* MC1061 cells were co-transformed with the two plasmids of interest and the transformants were selected by adding chloramphenicol and ampicillin to the solid medium. The presence of both fusions was confirmed by PCR and sequencing.

For β-galactosidase assays, the selected clones were grown in LB supplemented with ampicillin and chloramphenicol at 37°C until an OD_550_ of 0.2 was reached. IPTG was then added to the culture to a final concentration of 20 nM and the cultures were incubated at 37°C. Samples were taken 5 h after IPTG addition, and β-galactosidase activity was assayed as described by Miller (1991) [37]. The relative expression of β-galactosidase in each strain was calculated as the enzyme's activity with respect to that of the non-interaction control strain, which expressed the *Δ*αAmyA and *Δ*ωDnaE proteins [36]. The reported results are the means of at least three independent assays, each performed in triplicate.

### Construction of RecA and CheW tagged proteins

Co-immunoprecipitation assays were carried out using RecA-6×His and CheW-FLAG tagged proteins. The *recA* and *cheW* genes were PCR-amplified using the appropriate oligonucleotide pair (Table S1). In both cases, the corresponding tag sequence was included at the 5′ end of the suitable oligonucleotides that also contained a 3× Gly linker between the tag and the corresponding gene sequence (Table S1). The PCR products were digested with *Nde*I and *Bam*HI and cloned into pUA1108, with each tagged protein under the control of the Ptac promoter. The plasmids were transformed into *E. coli* DH5α and confirmed by sequencing. The confirmed plasmids were used in the electrotransformation of the *S.* Typhimurium *ΔrecA ΔcheW* strain, thereby ensuring that every RecA and CheW protein produced by that strain carried the specific tag. The selected transformants were confirmed again by PCR and sequencing.

### Co-immunoprecipitation assays

The assays were performed as described by D'Ulisse and others [38] with modifications. Briefly, cultures of *S.* Typhimurium *ΔrecA ΔcheW* harboring the plasmids with constructs encoding the tagged proteins were grown in LB broth supplemented with ampicillin to an OD_550_ of 0.2. Expression of the tagged genes was induced by the addition of 1 mM IPTG. After 3 h of growth, the cultures were centrifuged and the resulting pellet was washed in TBS 1× buffer (1.5 M NaCl, 250 mM Tris, pH 7.3) and then resuspended in cold IP lysis buffer (1× TBS, 15% glycerol, and 1% Triton X-100). The samples were incubated at 4°C for 40 min, with vortexing every 5 min. Finally, the samples were centrifuged at 4°C and the supernatant was collected. The protein concentration was determined by the Bradford method. As a control, cell lysates of *S.* Typhimurium *ΔrecA ΔcheW* containing the empty pUA1108 were also obtained following the same procedure.

Pure Proteome Protein A magnetic beads (Millipore) were used for immunoseparation. Either anti-6×His mouse IgG (Roche) or anti-FLAG mouse IgG (Acris) antibody was attached to the beads according to the manufacturer's instructions.

For co-immunoprecipitation the corresponding cell lysates were mixed at a ratio of 1∶1 and incubated at 30°C for 1 h without shaking, to allow interaction of the proteins. The appropriate antibody-coated magnetic beads (either anti-6×His or anti-FLAG IgGs) were added to the mixture and the samples were incubated overnight at 4°C with gentle mixing on a shaker. The beads were recovered and washed three times with wash buffer (1×TBS, 15% glycerol, and 1% Triton X-100) and finally resuspended in 45 µl of Laemmli sample buffer and heated for 10 min at 90°C. The samples were separated by SDS-PAGE on a 15% polyacrylamide gel and analyzed by western blotting using as primary antibody either anti-6×His or anti-FLAG mouse IgG and as secondary antibody horseradish peroxidase (HRP)-coupled anti-mouse IgG goat IgG antibody (Acris) together with Luminata western HRP chemiluminescence substrates (Millipore). HRP-coated Precision Plus protein western C standard (BioRad) was used as the molecular mass marker.

### Microfluidics assays

Microfluidics experiments were performed for each bacterial strain as previously described by Ahmed *et al.* [39]. After overnight incubation, cultures in log phase were diluted 1∶50, to a final volume of 2 mL, in fresh medium and grown to an optical density OD_600_ = 0.45±0.02. In order to optimize trajectory identification, cells were then diluted 1∶4 before injecting them in a 60 µm thick microfluidic channel hosting a microwell (∅/◯ 2 mm) at its center. The focal plane was set to 30 µm above the glass bottom, at channel mid-depth, to minimize the interaction of bacteria with surfaces. Cells were imaged with a 20× objective (Nikon Pan Fluor ELWD, NA 0.45, WD 7.4 mm) in phase contrast at 25 frames per second, for a total of 20 s per experiment. Three biological replicates, each with 5 technical replicates, were carried out for each strain. All frames were segmented to obtain the cells' coordinates and cells were then tracked using in-house developed tracking routines based on the nearest neighbor method, implemented in MATLAB (The Mathworks, Natick, MA). Manual selection of high-quality tracks followed trajectory identification and allowed 300–500 trajectories per strain to be used for the analysis of motility.

### Optical trapping assays

Optical trapping was carried out as previously described using a 1064-nm optical beam from a laser coupled to a single-mode fiber (Avanex) expanded up to 10 mm and then highly focused by an immersion oil objective (Nikon, CFI PL FL 100× NA 1.30 WD 0.16 mm) [29]. The oxygen scavenging system guaranteed a constant low level of oxygen and hence cell survival during the measurements [40].

As previously described [29], data for each bacterial strain were obtained from ten different randomly chosen cells of four distinct biological replicates; thus 40 cells per strain were analyzed.

The forward scattered light is collected by a 40X objective and projected into a quadrant photo diode (New Focus 2911). By this technique, the position of each trapped cell was acquired for 1000 s at 2 kHz of acquisition rate. The entire set of acquired data (1000 s) was then divided into 1-s-blocks. For each data block the angular velocity of the cell around the optical axis (Θ value) was calculated as described [29]. About 80% of the histograms showed very similar patterns. All plots shown below for the wild-type, mutant strains, and dead bacteria present the Θ histogram of one trapped cell either from the corresponding bacterial strain or from a dead cell control. In all cases, the selected histograms were within the above-mentioned 80%. During the experiments, videos were recorded using a CCD camera at the beginning (capture) and end (liberation) of the measurements.

### Chemotaxis capillary assays

Chemotaxis assays were conducted as described by Adler [30], with some modifications. The chemotaxis chamber set was formed by placing three V-shaped bent needles (40 mm 18 G needle, Nipro) on the surface of an aseptic 140-mm Petri dish (Deltalab) and then covering them with a 24×65 mm microscope cover slip (Menzel-Glässer).

One-ml capillary tubes, 3 cm long (Microcaps, Drummond Scientific Co.), were used. One end of each tube was heat-sealed in a flame. After autoclaving, the sealed capillaries were filled with either tethering buffer or 10 mM l-aspartate dissolved in tethering buffer [41].

Approximately 2 ml of each cell suspension was placed in the chemotaxis chambers, which were then incubated for 1 h at 30°C. After the incubation, the exterior of the capillaries was rinsed under a stream of sterile double-distilled water. The sealed end of the capillaries was then broken off and the contents of the tube were emptied into a 1.5-ml microcentrifuge tube containing 0.9% NaCl. Suitable dilutions were plated on LB plates; after an overnight incubation, the cfu/ml were calculated.

### Construction of the eYFP::*cheR* fusion

The enhanced yellow fluorescent protein (eYFP)::*cheR* fusion was constructed by the overlap extension procedure as follows. The *cheW* and *eYFP* genes (Clontech) were amplified using CheRstmF/CheRstmBamHI and eYFPNdeI/eYFPR oligonucleotide pairs, respectively, with eYFPR and cheRstmF containing complementary overhangs and a 3× Gly linker (Table S1). The resulting DNA fragments were annealed and amplified in a second round of PCR using eYFPNdeI and CheRstmBamHI to form the corresponding eYFP::*cheR* fusion. These outer primers contained *Nde*I and *Bam*HI restriction sites that were used to clone the fragments into the IPTG-inducible pUA1108 expression vector, giving rise to plasmid pUA1127, in which the eYFP::*cheR* fusion is under Plac promoter control. The fusion was confirmed by sequencing and the pUA1127 plasmid was transformed into several genetic backgrounds (*ΔcheR*, *ΔcheRΔrecA*, and *ΔcheRΔcheW*) to obtain the bacterial strains used in the chemoreceptor clustering assays.

### Chemoreceptor clustering assay

Receptor clustering experiments were performed as described [22], [42], [43] with modifications. Briefly, overnight cultures of *S.* Typhimurium strains carrying the pUA1127 (eYFP::*cheR*) plasmid were grown at 30°C in tryptone broth supplemented with ampicillin under constant agitation. After 24 h of incubation, the cultures were diluted 1∶100 in tryptone broth supplemented with ampicillin and 25 mM IPTG to induce eYFP::*cheR* fusion expression. The cultures were then incubated at 30°C until an OD_600_ of 0.5 was reached. The cells were harvested by low speed centrifugation (5300 g) for 15 min, washed once in cold tethering buffer, and finally resuspended in 100 ml of ice-cold tethering buffer. The cells were maintained on ice throughout the assay.

For fluorescence microscopy assays, the cells were immobilized and fixed at the same focal plane using thin 1% agarose pads in tethering buffer. Three µl of cells were applied on the pad, which was then covered with a clean cover slip. Fluorescence microscopy was performed using a Zeiss AxioImager M2 microscope (Carl Zeiss Microscopy) equipped with a Zeiss AxioCam MRm monochrome camera (Carl Zeiss Microscopy) and a filter set for eYFP (excitation BP 500/25; beam splitter FT 515; emission BP535/30). Cell fields were photographed and at least 250 cells were inspected by eye to determine the presence and type of clusters. All fluorescence images were obtained at 1000× magnification and were acquired under identical conditions. Each experiment was performed in triplicate using independent cultures. The images presented in the corresponding figure are representative of the entire images that are included as (Files S1, S2 and S3). ImageJ software (National Institutes of Health) was used to either quantify the number of clusters or to prepare images for publication.

### Statistical analysis

The chemotaxis capillary and chemotaxis clustering assays were statistically evaluated using, respectively, a two-way or one-way analysis of variance (ANOVA) with Prism (GraphPad), as previously described [44], [45]. In all cases, the analyses were followed by the Bonferroni multiple comparison post-hoc test, with p<0.05 defined as statistically significant. In all of the figures, the error bars indicate the standard deviation.

## Results

### The interaction of RecA and CheW proteins

Large-scale protein-protein *in vitro* interaction studies had previously identified RecA as a prey protein when CheW was used as bait, but not vice versa [23]. Thus, to ascertain the RecA and CheW interaction two-hybrid assays and co-immunoprecipitation experiments were carried out.

**Figure 1:**
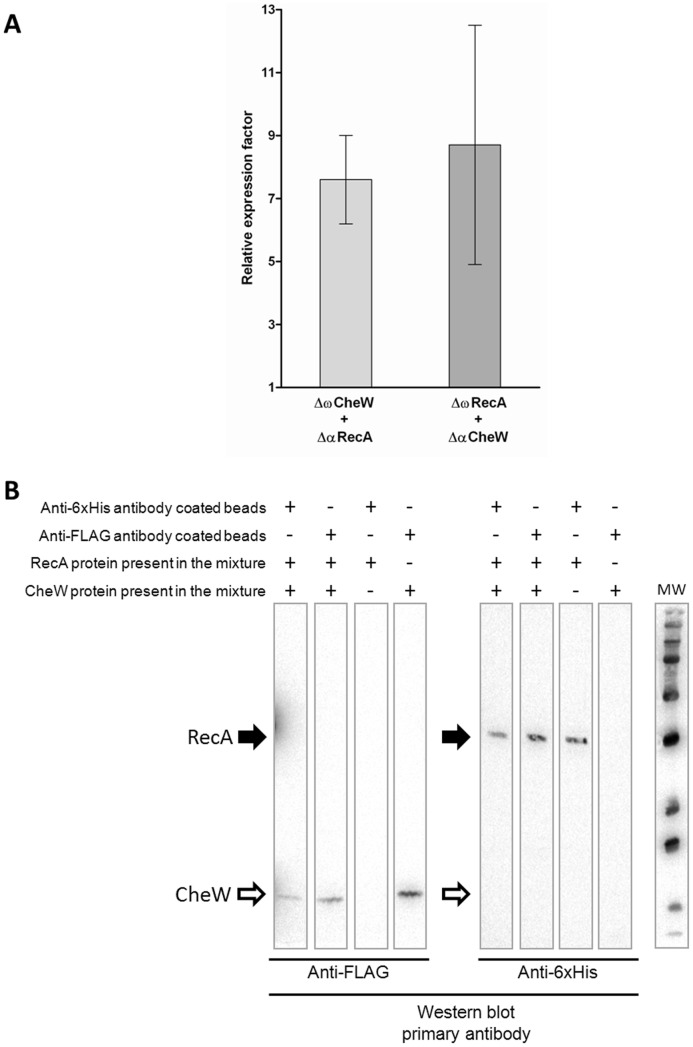
RecA protein directly interacts with CheW. **A) Two-hybrid assay.** Measurement of the β-galactosidase activity of strains co-expressing the chimera protein pairs *Δ*αRecA/*Δ*ωCheW or *Δ*αCheW/*Δ*ωRecA. The results are expressed relative to those obtained with the non-interacting control strain expressing *Δ*αAmyA and *Δ*ωDnaE [36]. Measurements were made 5 h after the addition of 20 nM IPTG to the culture. In each case the mean value from three independent experiments (performed in triplicate) is shown. Error bars indicate the standard deviation. **B) Co-immunoprecipitation assay.** Lysates prepared from cells overexpressing RecA-6×His and CheW-FLAG tagged proteins were mixed to allow the proteins to interact. Immunoprecipitation (IP) was performed by adding magnetic beads coated with either anti-6×His or anti-FLAG antibodies to the mixture and the attached proteins were recovered and separated by SDS-PAGE electrophoresis. The presence of the recombinant protein in the supernatants was assessed by western blotting (WB). As a control, co-IP assays were conducted using lysates from a *ΔrecAΔcheW S.* Typhimurium strain carrying an empty overexpression plasmid, thus expressing neither RecA-6×His nor CheW-FLAG proteins. The presence (+) or absence (−) of RecA, CheW, or both tagged proteins in the corresponding lysate mixture is indicated. Black and white arrows show the position of RecA-6×His and CheW-FLAG, respectively. IP indicates the antibody attached to the beads and WB the primary antibody used in western blotting. MW indicate the molecular mass marker.

In the two-hybrid assay, the previously described pB2H*Δ*α/pB2H*Δ*ω system [36] was used. RecA and CheW proteins were fused to the two non-functional but complementary β-galactosidase truncations (*Δ*α and *Δ*ω) in the system. In the reporter strain, β-galactosidase activity is driven by protein-protein recognition between both non-β-galactosidase parts of the chimeras. As shown in Fig. 1A, relative β-galactosidase expression by strains co-expressing either the *Δ*αRecA/*Δ*ωCheW or the *Δ*αCheW/*Δ*ωRecA chimera pair was >7, indicating significantly higher β-galactosidase activity in these strains than in the non-interaction assay control strain [36].

To further confirm the two-hybrid assay results and thus obtain additional evidence for the interaction between RecA and CheW, co-immunoprecipitation assays were carried out using *S.* Typhimurium *ΔrecA ΔcheW* strains carrying the corresponding plasmids that overexpress either the RecA-6×His or the CheW-FLAG tagged proteins (Table 1). The immunoprecipitation was performed by using magnetic beads coated with either anti-6×His or anti-FLAG mouse IgG antibodies that specifically interact with the corresponding tagged protein and the recovered proteins were detected by Western blot. As seen in Fig. 1B, when both recombinant proteins were present in the protein mixture, and anti-6×His antibody coated beads were used, CheW-FLAG proteins were observed in the recovered supernatants. The same results were observed when anti-FLAG antibody coated beads were added to the mixture, then RecA-6×His proteins were recovered. All together, these data indicate that RecA-6×His was able to pull down CheW-FLAG and vice versa. Thus, the results of the two assays together confirm RecA–CheW pair formation and suggest the association of RecA with the chemotaxis pathway through its interaction with CheW.

### The absence of RecA causes a decrease in swimming speed

**Figure 2:**
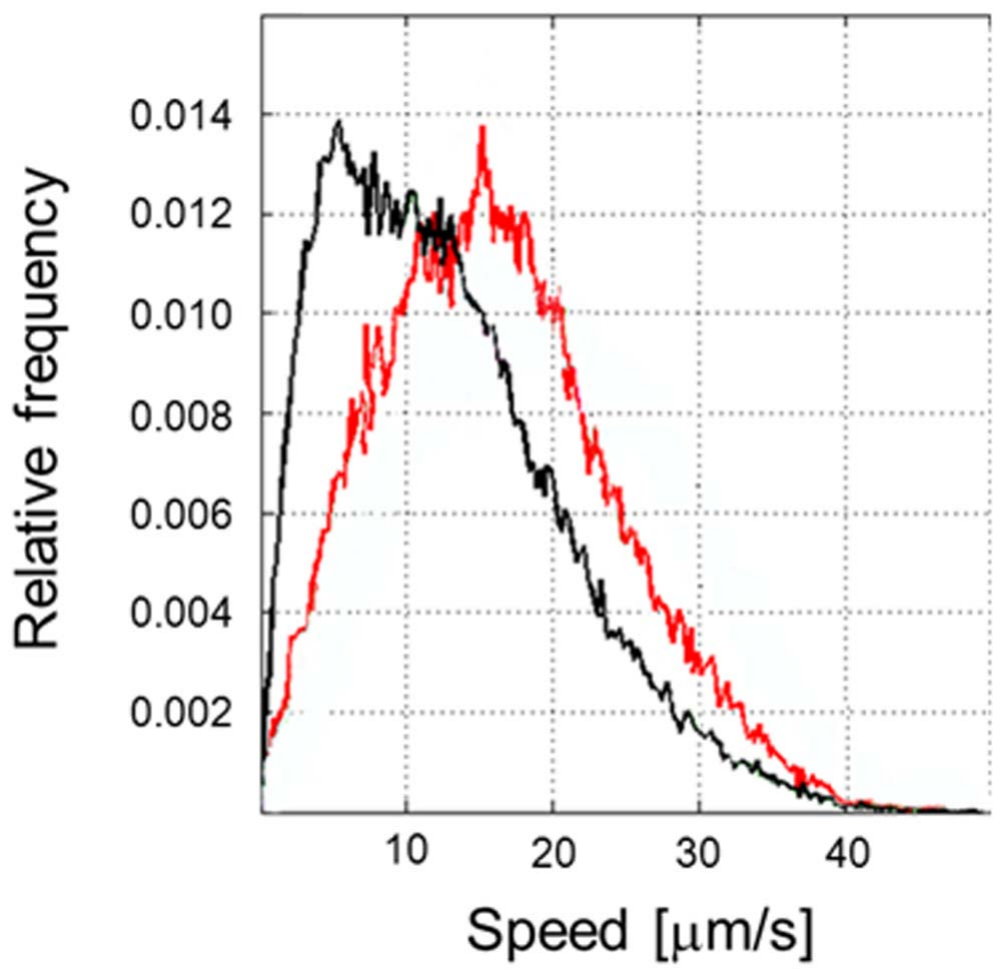
The lack of *recA* reduces the swimming speed of *S*. Typhimurium. The experimentally observed probability distribution of the swimming speeds within a population of wild-type *S*. Typhimurium (red line) and of the *ΔrecA* mutant (black line), assessed using a microfluidics assay [39]. Values are expressed as the relative frequency of a given speed within a cell population. For each strain, the results were obtained from three independent experiments supported by five technical replicates each, for a total of 300–500 cells tracked per strain.

To further understand the role of RecA in motility, the swimming speed of a *S.* Typhimurium Δ*recA* mutant was evaluated through microfluidics assays and compared to that of the wild-type strain. In this assay, swimming speed computed over the whole set of identified trajectories was measured. As shown in Fig. 2, which depicts the relative frequency of swimming speeds for each strain, the absence of RecA prompted a change in the swimming profile. Specifically, at the highest relative frequency, the velocity of the mutant was lower than that of the wild-type strain under the same experimental conditions.

### The *ΔrecA* mutant present a CW-bias of flagella rotation

To determine whether the slower swimming speed prompted by the absence of RecA was due to a bias in flagellar rotation, the flagellar rotation patterns of the *ΔrecA* mutant was studied using a single optical trap. Optical trapping is an excellent tool for analyzing the dynamic properties of bacteria [46]–[48]. It is based on the ability of an optical beam to trap a single cell because the refractive index of cells (and their constituents) is higher than that of the surrounding medium. Once trapped, the movement of that cell is measured, yielding information on its momentary position. In the case of rod shaped *S.* Typhimurium, a cell trapped in the single optical beam aligns itself along the optical axis. Thus, besides Brownian motion, the torque produced by flagellar rotation alters the dynamics of the cell [29]. The measurement of the rotation profile for each strain is expressed as the distribution of the change in the mean value of Θ, which is the angular velocity of the cell around the optical axis [29]. Using this technique we were able to distinguish CW to CCW switching of the flagella.

**Figure 3:**
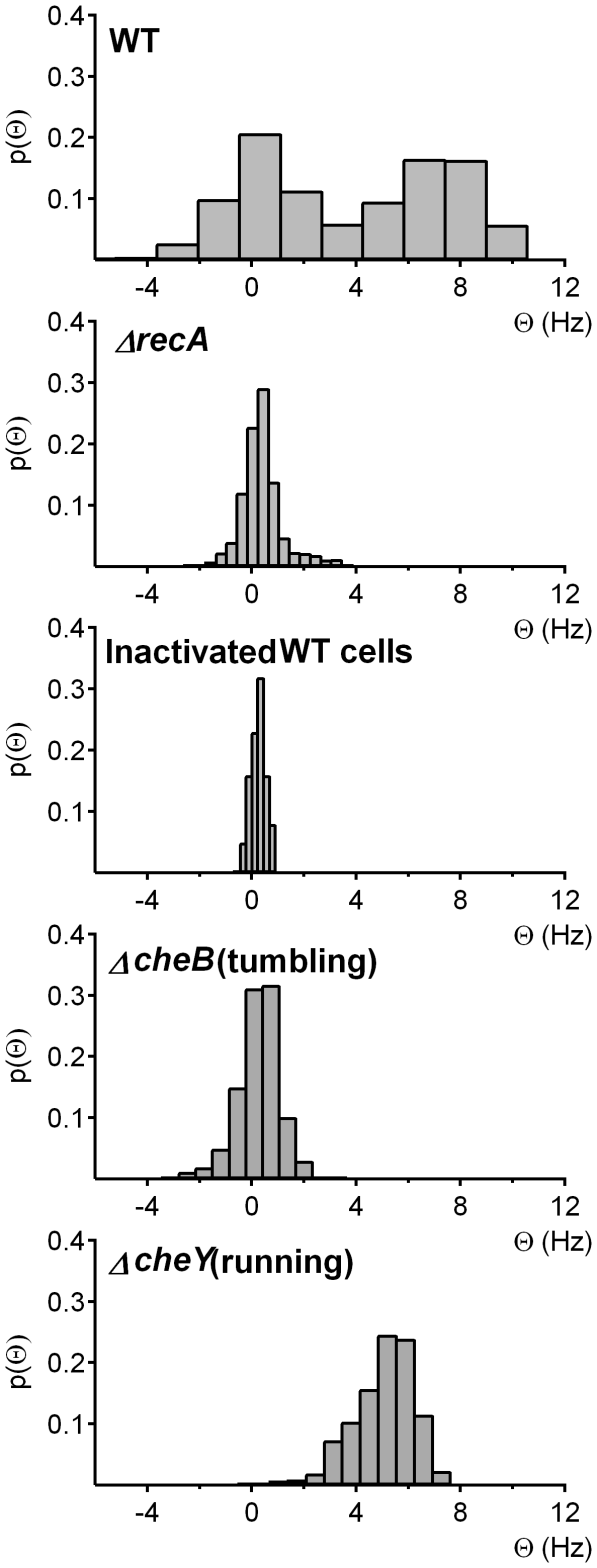
Flagellar rotation is CW-biased in the *S*. Typhimurium Δ*recA* mutant. The flagellar switching profiles of *S*. Typhimurium LT2 wild-type (WT), dead wild-type (dead), *ΔrecA*, *ΔcheB* (tumbling), and *ΔcheY* (running) cells were evaluated. The resulting histograms show the distribution of the change in the mean cellular angular velocity around the optical axis (Θ). A zero-centered peak, as displayed by dead cells and the *ΔcheB* tumbling mutant, is indicative of CW-biased flagellar rotation, and a peak with positive values, as displayed by the *ΔcheY* running mutant, CCW-biased rotation. The presence of two peaks, one zero-centered and the other centered at positive values, indicates a mixed population displaying both CW and CCW rotational patterns and thus a non-biased flagellar rotational pattern. For each strain, the results were obtained from four independent experiments of ten cells each.

Figure 3 shows the flagellar rotation profile of both the *S.* Typhimurium *ΔrecA* mutant and the wild-type strain. Dead wild-type cells and *ΔcheB* and *ΔcheY* mutants were used as controls. There is no change in the angular velocity of dead cells, which exhibit only Brownian motion; thus, their Θ distribution pattern is centered at zero. Among the mutants, Δ*cheB* cells, described by their tumbling motility because of their CW flagellar rotation bias [49], displayed a Θ distribution pattern centered near zero. Thus, there was no change in the average angular velocity of the mutant cells but, as would be expected, the histogram was broader than that of the dead cells. Conversely, the flagella rotation profile of *ΔcheY* cells had a mean Θ that was not zero and was highly positive, consistent with the smooth swimming ability characteristic of this strain because of its CCW-biased flagellar rotation pattern [50], [51]. In the living wild-type cells, the Θ histogram showed the anticipated two peaks, reflecting normal switching between CCW and CW flagellar rotations. The peak centered at zero along the *x* axis corresponded to the tumbling state (CW rotation) whereas the peak located around 7.5 was due to the running state (CCW rotation) (Fig. 3). By contrast, but similar to the *ΔcheB* mutant, *ΔrecA* cells showed only one peak, centered near zero, in their Θ distribution pattern (Fig. 3). The absence of the second peak was indicative of the CW-biased rotation of Δ*recA* cells.

### Chemotaxis response of *recA* mutants

**Figure 4:**
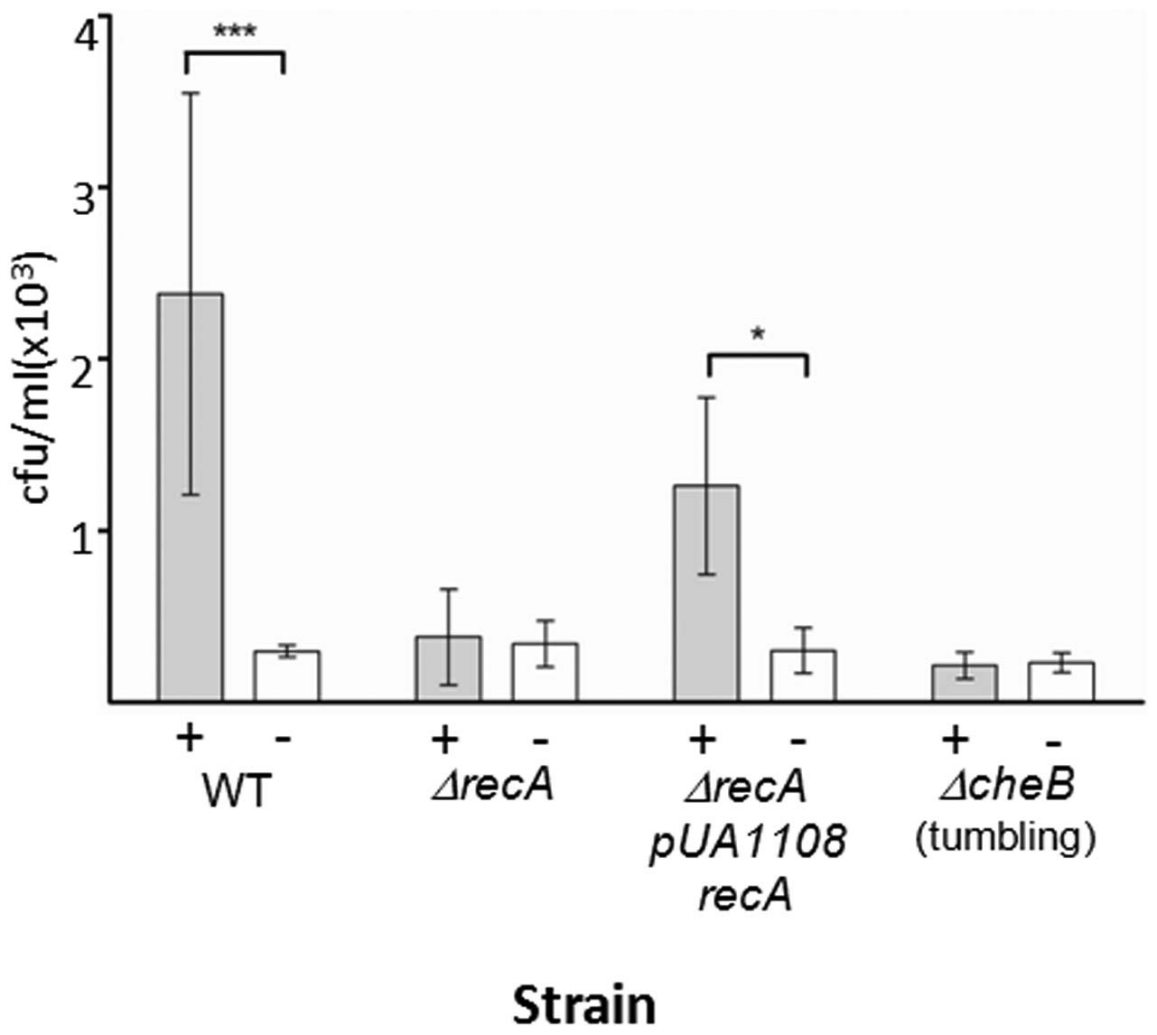
The chemotactic response of the *S*. Typhimurium Δ*recA* mutant is impaired. The chemotactic responses of *S*. Typhimurium wild-type (WT), Δ*recA*, Δ*recA* complemented (Δ*recA* pUA1109), and Δ*cheB* (tumbling) cells were assessed using Adler's capillary assay [30] with the modifications described in Materials and Methods. Values are expressed as the number of viable cells (in cfu/ml) in a capillary tube containing either 10 mM aspartate (+) or tethering buffer alone (−). The results are the mean of five independent experiments of three capillaries each. Error bars indicate the standard deviation. ***p<0.001 and *p<0.05 as determined by two-way ANOVA with Bonferroni correction.

To further confirm the switching defect of the *ΔrecA* mutant, its ability to move towards an l-aspartate source was evaluated using a classical capillary assay. The results are shown in Fig. 4. As expected, and in concordance with observations in other tumbling strains such as the *ΔcheB* mutant [49], the capillary assays clearly demonstrated that cells lacking RecA are unable to respond to the presence of l-aspartate. Furthermore, chemotaxis by the *ΔrecA* mutant was restored when the RecA deficiency was complemented by the presence of a plasmid containing the *recA* gene under the control of an IPTG- inducible promoter (Fig. 4) but not by the presence of the empty plasmid (data not shown). These results unequivocally showed that the presence of RecA is essential for a normal chemotactic response.

### Chemotaxis receptor clustering

Based on our results, the phenotype of the *ΔrecA* mutant is similar to that of other *che* mutants, since in all cases the absence of RecA impairs not only swarming [17], [18] but also the switching of flagellar rotation (Fig. 3) and the chemotactic response (Fig. 4). To elucidate the role of RecA in chemotaxis, and taking into consideration the direct interaction of RecA with the CheW coupling protein, which bridges the MCPs to histidine kinase CheA [43], [52], we asked whether, like CheW, RecA was involved in chemoreceptor clustering. To investigate this possibility fluorescently tagged CheR (eYFP-CheR) was used as a specific reporter for chemoreceptor localization [43].

**Figure 5:**
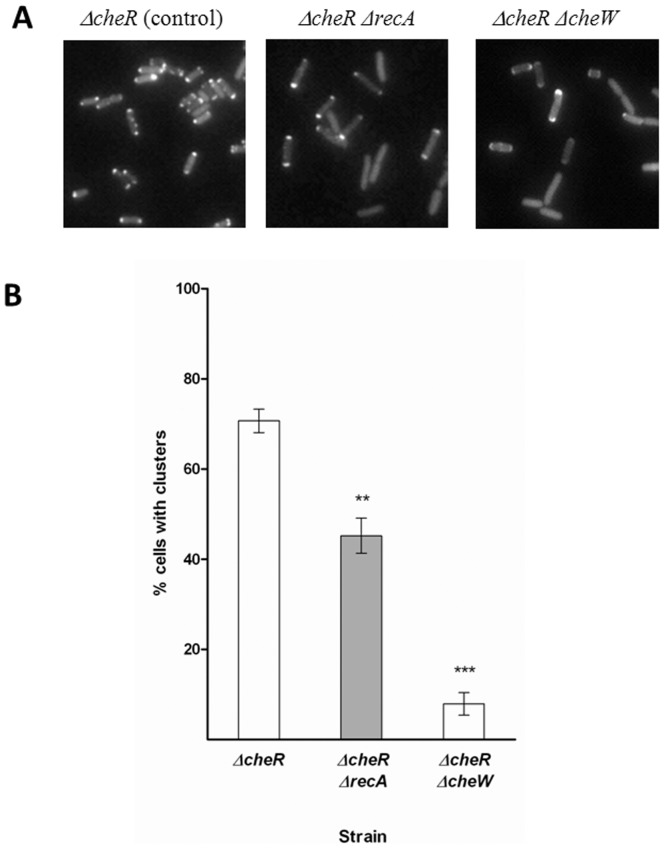
The formation of polar chemoreceptor clusters is altered in the absence of RecA protein. **A**) A representative fluorescence microscopy image of the *ΔcheR ΔrecA* strain harboring plasmid pUA1127, containing the inducible eYFP::*cheR* fusion. Images of the *ΔcheR* and *ΔcheRΔcheW* strains containing the gene fusion were also included as positive and negative controls of polar chemoreceptor cluster structuring, respectively. **B**) The fraction of cells with well-structured polar chemoreceptor clusters. The percentage of cells showing polar, round, and diffraction-limited spots (previously referred to as clusters; [43]) was quantified in each strain. The results are the mean of three independent imaging experiments. Error bars represent standard deviation. ***p<0.001 and *p<0.01 as determined by one-way ANOVA with Bonferroni correction.

The eYFP-CheR fusion was constructed and cloned into pUA1108 vector under the control of an IPTG-inducible promoter (Table 1). For correct chemoreceptor localization, native CheR had to be removed; accordingly, the plasmid was included in the *S.* Typhimurium *ΔcheR ΔrecA* transformant. Additionally, it was also used to obtain the *ΔcheR* and *ΔcheR ΔcheW* mutants. The *ΔcheR* mutant served as the positive control strain since it exhibited normal polar clusters. The *ΔcheR ΔcheW* strain was used as the negative control strain since the absence of CheW inhibits polar cluster formation [22]. As expected, and in agreement with previous reports [43], in the positive control (the *ΔcheR* strain) single tight polar spots were seen in ∼70% of the observed cells. These spots corresponded to the clustering of thousands of chemoreceptors at the cell pole (Fig. 5). However, in agreement with previous data [43], in the absence of CheW compact polar clusters were formed in only ∼10% of the cells; instead, the presence of diffuse clusters (known as caps) was observed (Fig. 5A). Thus, according to our findings, the absence of RecA significantly impairs normal polar cluster formation, which occurred in only ∼50% of the cells, and increases the presence of caps. Nonetheless, neither the reduction in polar spot formation nor the increase in caps was as high in the absence of RecA as in the absence of CheW.

## Discussion

A role for RecA in controlling the swarming motility of both *E. coli* and *S*. Typhimurium was clearly shown in previous studies [17], [18]. However, besides its possible connection to CheW [18], [23], nothing was known about the mechanisms that link RecA to motility. To determine whether RecA is associated with the chemotaxis pathway and, specifically, with flagellar function, we examined its putative direct interaction with CheW, its role in swimming motility and in chemotaxis, as well as the flagellar switching pattern of cells lacking RecA.

Our results support a tight relationship between RecA and the chemotactic response. First, our results unequivocally confirmed the interaction of RecA with CheW through two widely used techniques. The results of the two-hybrid assays were in concordance with those previously obtained in a large-scale genome-wide screening assay [23] and suggested an interaction between RecA and CheW (Fig. 1A), which was definitively shown in the co-immunoprecipitation assays (Fig. 1B). Second, the motility phenotype of cells lacking RecA, as determined herein, was similar to that of some *che* mutants. The latter finding was supported by microfluidics assays, in which the average swimming speed of *S.* Typhimurium *ΔrecA* was lower than that of the wild-type strain (Fig. 2), and by the observed differences in the flagellar rotation patterns of these two strains (Fig. 3). Thus, the absence of RecA impaired flagellar switching, leading to a CW bias similar to that of other tumbling strains, like the *ΔcheB* mutants [49]. Furthermore, consistent with the tumbling phenotype of the *ΔrecA* mutant, our results demonstrate that RecA is essential for a normal chemotactic response. Specifically, in quantitative chemotaxis assays the *ΔrecA* mutant was unable to detect the presence of l-aspartate, a well-known chemoattractant (Fig. 4); instead, chemotaxis was restored only when the *recA* deficiency was complemented by a plasmid carrying a copy of the *recA* gene (Fig. 4). The slower-moving phenotype of the RecA-deficient mutants can be explained by the CW bias displayed by these cells. By being anchored in a tumbling state, without normal running, the *ΔrecA* mutant was slower than the wild-type strain. In a previous study, the inability to switch the direction of flagellar rotation was linked to defects in chemotaxis and to improper colony hydration, leading to an inability to swarm [25], [27], [28]. It was previously reported that an *E. coli recA1* mutant did not exhibit any apparent alterations in chemotaxis [53]. Nevertheless, this *recA1* strain was not a knockout mutant, as was the *ΔrecA* mutant used in this work, in which the *recA* gene was completely removed. Furthermore, the *recA1* allele is a single amino acid missense mutation that prevents RecA recombinatorial activity [54] but still allows normal binding to ssDNA as well as ATP-independent renaturation of complementary ssDNA molecules [55]. Thus, the results obtained with the two mutants cannot be compared.

Nevertheless, how RecA modulates flagellar rotation was unclear. In an earlier study, the absence of RecA had no effect on the expression of genes involved in either flagellar biosynthesis or chemotaxis [17], as shown for other proteins such as H-NS, which is required not only for flagellar motor function but also for flagellar biogenesis [56]. The direct association of RecA with the CheW coupling protein led us to ask whether RecA, like CheW, plays a role in the architecture of chemoreceptor arrays. CheW tethers CheA kinase to the MCPs forming the MCP-CheW-CheA ternary complexes and chemoreceptor arrays, enabling MCPs to modulate CheA autokinase activity [22], [57] which, in turn, controls the level of phosphorylated CheY (CheY-P). Once activated by the MCPs, phosphorylated CheA transfers its phosphoryl group to CheY. CheY-P then promotes a switch in the direction of flagellar rotation, from CCW to CW. According to our observations, the RecA protein is necessary for the formation of normal polar chemoreceptors arrays. Although its role may not be the same as that of CheW, the absence of RecA significantly reduces the polar clustering of chemoreceptors (Fig. 5). The absence of MCPs, CheW, or CheA is known to impair chemoreceptor array formation, leading cells to run constantly because of the CCW bias of their flagellar rotation [51]. Conversely, and in addition to the demonstrated effect of RecA on chemoreceptor array formation, our results show that the absence of this protein results in a CW bias, similar to that observed in *cheZ, cheR*, or *cheB* null mutants. All of these Che proteins are associated with chemotactic response adaptation: CheZ phosphorylase returns phosphorylated Che (CheY-P) to its non-phosphorylated state (CheY), and CheB methylesterase and CheR methyltransferase control the MCP methylation state, adjusting it to the presence and concentration of external stimuli. Therefore, a similar function can be hypothesized for RecA in the chemotactic response adaptation. It is worth noting that although CheZ, CheR, and CheB co-localize with MCPs-CheW-CheA complexes at the cell poles [42], none of these proteins have an effect on polar cluster formation [43], unlike RecA. While further work is needed to determine the exact role of RecA in the chemotaxis pathway, our results clearly reveal previously unknown functions of RecA: its involvement in the control or modulation of flagellar rotation, and thus not only with swarming but also with swimming and chemotaxis, and its role in the architecture of polar chemoreceptor arrays.

## Supporting Information

Table S1 **Oligonucleotides used in this work.**
(DOCX)

File S1 **ZIP File containing the entire set of images used to determine the presence and type of clusters found in the** ***S.*** **Typhimurium UA1910 (** ***ΔcheR*** **) strain carrying the pUA1127 (eYFP::** ***cheR*** **) plasmid.**
(RAR)

File S2 **ZIP File containing the entire set of images used to determine the presence and type of clusters found in the** ***S.*** **Typhimurium UA1915 (** ***ΔcheRΔcheW*** **) strain carrying the pUA1127 (eYFP::** ***cheR*** **) plasmid.**
(RAR)

File S3 **ZIP File containing the entire set of images used to determine the presence and type of clusters found in the** ***S.*** **Typhimurium UA1913 (** ***ΔcheRΔrecA*** **) strain carrying the pUA1127 (eYFP::** ***cheR*** **) plasmid.**
(RAR)
