# Supplementary material for: RecA Protein Plays a Role in the Chemotactic Response and Chemoreceptor Clustering of Salmonella enterica
Source: PLoS One. 2014 Aug 22;9(8):e105578. doi: 10.1371/journal.pone.0105578 (PMC4141790; doi:10.1371/journal.pone.0105578)
Supplement: Table S1 — Oligonucleotides used in this work. (DOCX) [file pone.0105578.s001.docx]

**Table S1. Oligonucleotides used in this work**

| Name | Sequence (5’→ 3’) ^a^ | Application |
| --- | --- | --- |
| P1recA ^a^ | GAAAGCGTTGGCGGCAGCACTGGGCCAAATTGAAAAGCAATTTGGTAAAGGCTCCATCATGCGTCTGGGTGAAGACCGTTgtgtaggctggagctgcttc | P1 primer used for *S.* Typhimurium *ΔrecA* mutant construction |
| P2recA ^a^ | CAACGCCTTCGCTATCGTCAACGGCGAAATCGGGCGTGGCATTCTGATTACTCAACAGTAATTCACGAACCCTTTTCTCGatgggaattagccatggtcc | P2 primer used for *S.* Typhimurium *ΔrecA* mutant construction |
| P1cheW ^a^ | GGCGAGCCGTCAGGTCAGGAATTCCTGGTGTTTACACTGGGAAATGAAGAGTACGGCATCGATATCCTGAAAGTGCAGGA*gtgtaggctggagctgcttc* | P1 primer used for *S.* Typhimurium Δ*cheW* mutant construction |
| P2cheW ^a^ | TATCCAGCAGCGCCATCTCTTCGCTGTTAAGCAGTTTTTCGATATTCACCAGAATCAGCATACGCTCGCCGAGCGCGCCC*atgggaattagccatggtcc* | P2 primer used for *S.* Typhimurium *cheW* mutants construction |
| P1cheR ^a^ | CATCTCTGCCCTCCGGGCAAACGTCAGTATTGTTACAGATGACACAGCGCCTCGCGCTGTCCGACGCGCATTTTCGTCGG*gtgtaggctggagctgcttc* | P1 primer used for *S.* Typhimurium Δ*cheR* mutant construction |
| P2cheR^a^ | CTTACTTAGCGCATACACCGTCTGTCCGCGCAGGCTAAACTCGCGCACGAGGTTGCTAAAGTTTTCTGAGTGACCGGCAA*atgggaattagccatggtcc* | P2 primer used for *S.* Typhimurium *cheR* mutant construction |
| RecAextup | CAGTGGAGAAGGGATTACGC | Upper primer used for *S.* Typhimurium *ΔrecA* mutant confirmation by PCR and sequencing |
| RecAextdw | TGGAGCCTTTACCAAATTGC | Lower primer used for *S*. Typhimurium *ΔrecA* mutant confirmation by PCR and sequencing |
| CheWeF | GTCACGTTGAGATCCAGTCA | Upper primer used for *S.* Typhimurium cheW mutants confirmation by PCR and sequencing |
| CheWeR | TCGCTGGCAATGGCGTCATA | Lower primer used for *S.* Typhimurium *cheW* mutants confirmation by PCR and sequencing |
| CheRextF | CAGTCATTAGCCGCCAGGGA | Upper primer used for *S.* Typhimurium cheW mutants confirmation by PCR and sequencing |
| CheRextR | TCGGAACCTTTCCCGGTCAG | Lower primer used for *S.* Typhimurium *cheW* mutants confirmation by PCR and sequencing |
| recA-SphI^b^ | gcatgcATGGCTATCGACGAAAACAA | Upper primer used for *recA* clonning into pB2HΔα and pB2HΔω vectors |
| recA-BamHI^b^ | ggatccAAAATCTTCGTTGGTTTCTG | Lower primer used for *recA* clonning into pB2HΔα and pB2HΔω vectors |
| cheW-SphI^b^ | gcatgcATGACCGGTATGAGTAATGT | Upper primer used for *cheW* clonning into pB2HΔα and pB2HΔω vectors |
| cheW-BamHI^b^ | ggatccCGCGACGTGTGATGCTGCGA | Lower primer used for *cheW* clonning into pB2HΔα and pB2HΔω vectors |
| amyA-SphI^b^ | gcatgcATGAAAAACCCCACGTTATT | Upper primer used for *amyA* clonning into B2HΔα vector |
| amyA-BamHI^b^ | ggatccCACGTCCTCAATGACCCACA | Lower primer used for *amyA* clonning into B2HΔα vector |
| dnaE-SphI^b^ | gcatgcATGTCTGAACCACGTTTCGT | Upper primer used for *dnaE* clonning into pB2HΔω vector |
| dnaE-BamHI^b^ | ggatccGTCAAACTCCAGTTCCACCT | Lower primer used for *dnaE* clonning into B2HΔω vector |
| RecANdeF^b^ | GGAATTCcatatgGCTATCGACGAAAACAAACAG | Upper primer used for RecA-6xHis clonning |
| recABamFLr^b,c^ | CGggatccTTAATGATGATGATGATGATG***GCCGCCGCC***AAAATCTTCGTTGGTTTCTG | Lower primer used for RecA-6xHis clonning |
| cheW-Nde-f^b^ | GGAATTCcatatgACCGGTATGAGTAATGTAAGC | Upper primer used for CheW-FLAG clonning |
| cheWBamHIr^b,c^ | CGggatccTTATTTGTCGTCGTCGTCTTTGTAGTC***GCCGCCGCC***CGCGACGTGTGATGCTGCGATATCC | Lower primer used for CheW-FLAG clonning |

^a^ P1 and P2 sequences, homologues to the pKD3 plasmid, are represented in lower case italics.

^b^ Restriction Endonuclease sites are indicated in lower case.

^C^ 6xHis or FLAG tags are underlined and Gly spacers are in bold and italics.
